# Adequate Placental Sampling for the Diagnosis and Characterization of Placental Infection by Zika Virus

**DOI:** 10.3389/fmicb.2020.00112

**Published:** 2020-02-21

**Authors:** Emanuella Meneses Venceslau, José Paulo Siqueira Guida, Guilherme de Moraes Nobrega, Ana Paula Samogim, Pierina Lorencini Parise, Rodolfo Rosa Japecanga, Daniel Augusto de Toledo-Teixeira, Julia Forato, Arthur Antolini-Tavares, Arethusa Souza, Albina Altemani, Silvio Roberto Consonni, Renato Passini, Eliana Amaral, Jose Luiz Proenca-Modena, Maria Laura Costa, The Zika-Unicamp Network, John D. Horowitz

**Affiliations:** Faculty of Food Engineering, University of Campinas, Campinas, Brazil; Department of Obstetrics and Gynecology, School of Medical Sciences, University of Campinas, Campinas, Brazil; Clinical Pathology Department, School of Medical Sciences, University of Campinas, Campinas, Brazil; Pediatric Immunology, Center for Investigation in Pediatrics, Faculty of Medical Sciences, University of Campinas, Campinas, Brazil; Department of Human Development and Rehabilitation, Faculty of Medical Sciences, University of Campinas, Campinas, Brazil; Neurology Department, Faculty of Medical Sciences, University of Campinas, Campinas, Brazil; Campinas Department of Public Health Surveillance, Campinas, Brazil; Department of Genetics, Evolution, Microbiology and Immunology, Institute of Biology, University of Campinas, Campinas, Brazil; School of Pharmaceutical Sciences, University of Campinas, Campinas, Brazil; Department of Genetics, Evolution, Microbiology and Immunology, Institute of Biology, University of Campinas, Campinas, Brazil; ^1^Department of Obstetrics and Gynecology, School of Medical Sciences, University of Campinas, Campinas, Brazil; ^2^Department of Genetics, Evolution, Microbiology and Immunology, Institute of Biology, University of Campinas, Campinas, Brazil; ^3^Department of Pathological Anatomy, School of Medical Sciences, University of Campinas, Campinas, Brazil; ^4^Department of Biochemistry and Tissue Biology, Institute of Biology, University of Campinas, Campinas, Brazil

**Keywords:** Zika virus, placenta, viral persistence, pregnancy, systematic sampling, qRT-PCR

## Abstract

The detection of Zika virus (ZIKV) in immunoprivileged anatomical sites, potential sites for viral persistence, may guide the confirmation of undefined cases of ZIKV infection and also bring to light unknown pathways of viral transmission. Thus, this study aimed to characterize ZIKV infection in stratified, standardized placental samples in women with exanthematic febrile manifestations during pregnancy and compare findings to the standard investigation protocol of official health agencies. To this end, a case series of placental findings within a prospective cohort study was conducted over a period of 24 months. Serum/urine were obtained at the time of clinical case identification. Placental sampling was performed following standard investigation protocol (samples of 1.0 cm sent to a reference laboratory) and in a systematic way at various regions, such as chorionic plate, chorionic villi, basal plate, amniotic membrane, and umbilical cord, for subsequent ZIKV identification and quantification. Clinical information was obtained and histological preparation with hematoxylin–eosin staining for morphological evaluation was performed. This case series included 17 placentas systematically collected. Of these, 14 were positive by qRT-PCR for ZIKV, 5 in the umbilical cord, 7 in the amniotic membrane, 7 in the chorionic plate, 13 in the chorionic villi, and 7 in the basal plate, whereas none were reported by the reference laboratory. The most common morphological and anatomopathological findings were increased stromal cellularity, villitis, calcification, maternal vascular malperfusion, placental hypoplasia, and maternal–fetal hemorrhage (intervillous thrombi). Seven women presented positive testing for ZIKV in serological and/or molecular tests during gestation in urine. While viral quantification in urine ranged from 10^1^ to 10^3^ FFU eq/ml, that in different placental regions ranged from 10^3^ to 10^8^ FFU eq/g. Thus, ZIKV can infect different regions of the placenta and umbilical cord of pregnant women, showing that the systematic collection and adequate storage of the placenta is fundamental for the detection of ZIKV in this organ. The detection of ZIKV in the placenta after several months of initial symptoms suggests that this tissue may be a site for viral persistence during pregnancy.

## Introduction

The introduction of the Zika virus (ZIKV) in Brazil between 2013 and 2014 revealed previously unknown clinical–pathogenic aspects that had great repercussion in the medical community worldwide. ZIKV, previously described as a virus associated with the development of self-limited acute exanthematous and febrile disease with rare case reports of suspected neurologic sequelae, such as Guillain-Barré syndrome, was suddenly shown to cause severe clinical complications (Oehler et al., 2014).

Particularly in pregnant women, it could lead to fetal malformations, further detailed as congenital Zika virus syndrome (CZS). This syndrome is characterized by a broad spectrum of fetal changes, with microcephaly being the most striking, even if present only in part of the affected newborns (Petersen et al., 2016; Rasmussen et al., 2016; van der Eijk et al., 2016; Ministério da Saúde, 2017). Clinical consequences of CZS include the presence of pronounced brain damage and intracerebral calcification, cortical reduction, atrophy, cerebellar hypoplasia, arthrogryposis, ocular lesions, and neurogenic bladder (Chimelli et al., 2017; Honein et al., 2017; Costa Monteiro et al., 2018). In Brazil, 2865 cases of newborns and children with growth alterations and microcephaly caused by Zika infection during pregnancy were reported from November 8, 2015, to December 29, 2018, not counting those who died (Ministério da Saúde, 2019).

Zika virus are enveloped viruses, with a positive-sense single-stranded RNA genome belonging to the *Flaviviridae* family (Simmonds et al., 2017) transmitted mainly by arthropod bites, such as the *Aedes aegypti* mosquito in an urban environment, or less frequently by sexual intercourse, transfusion, or vertically from the mother to the fetus during pregnancy or childbirth (D’Ortenzio et al., 2016; Driggers et al., 2016; Turmel et al., 2016).

Acute ZIKV infection is often asymptomatic, which makes it difficult to confirm the infection in pregnant women with fetal alterations and without reports of febrile exanthematous disease during the acute phase. Also, the serological tests available on the market have high cross-reactivity with other arboviruses, such as dengue and yellow fever. To evaluate conflicting serological results by ELISA, there are plaque reduction neutralization tests (PRNT) that can help to determine cross-reactivity between flaviviruses (Sharp et al., 2019). In addition, the window for detection of viral RNA in blood and urine is relatively short, even in symptomatic patients, making the laboratory diagnosis particularly difficult in a significant proportion of infected patients (Xavier et al., 2017). Thus, detection of ZIKV in immunoprivileged anatomical sites, such as the placenta, which could be sites of viral persistence, may be fundamental for the confirmation of undefined cases of ZIKV infection.

Currently in Brazil, laboratory diagnosis of the presence of ZIKV in placental tissue is performed by reference laboratories of the Unified Health System (SUS) network using real-time polymerase chain reaction after reverse transcriptase reaction (qRT-PCR) from placental biopsies of 1.0 × 1.0 cm, without any other requirement detail (Ministério da Saúde, 2015). In addition, protocols for sampling and placental storage differ across the world (Wolfe et al., 2014).

Thus, in this study, we evaluated the results and importance of a stratified, standardized sampling and storage composed of four biopsies containing different placental layers, collected equidistant from the umbilical cord insertion, for an accurate diagnosis of ZIKV in placentas of women with exanthematous febrile manifestations during pregnancy. All the data obtained were compared with the results of ZIKV in the placenta of these same pregnant women provided by the reference laboratory for detection of ZIKV. The present study further characterizes viral load and provides an anatomopathological examination.

## Materials and Methods

### Patients

**FIGURE 1:**
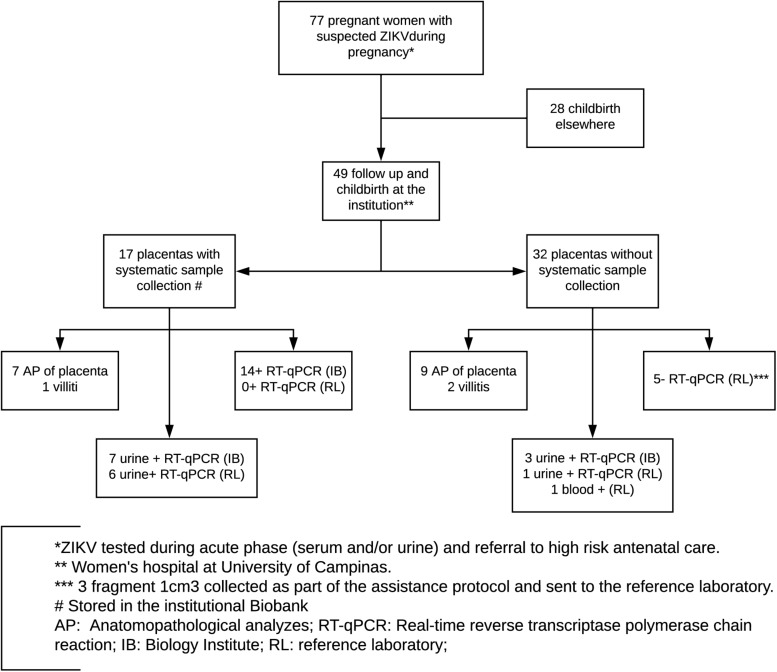
Flowchart with included women in the considered cohort, diagnostic testing, and follow-up.

In the present prospective cohort study, 77 pregnant women were referred to the Women’s Hospital at the University of Campinas from February 2016 to January 2018 presenting symptoms that suggested arbovirus infection. These symptoms included maculopapular rash associated or not with conjunctival hyperemia without secretion and without pruritus, fever, polyarthralgia, and periarticular edema. In addition, women who presented with sudden onset fever (≥38.5°C) and arthralgia or acute arthritis with unexplained acute onset by other conditions; women with fever with no defined etiology associated with myalgia, headache, prostration, and retroorbital pain; and those with identification of fetal microcephaly by ultrasonographic examination were also included. All pregnant women included were followed until childbirth and were tested also for HIV, hepatitis B, hepatitis C, syphilis, toxoplasma, cytomegalovirus, rubella, and Epstein–Barr virus by serological tests or conventional PCR. None of the considered cases were positive for such pathogens. Of the 77 pregnant women referred, 49 had childbirths in the University of Campinas, from which 17 placentas were sampled according to a systematic protocol standardized in this study, depending on the identification of cases by the team responsible for medical care at the time and adequate storage and communication to the technical staff trained for placental sampling. These 17 cases represent a case series within the cohort with further detailed evaluation of viral detection. Conditions such as childbirth time, allocated medical staff, and general instructions of placenta destination influenced which women had their placentas collected (Figure 1). In addition, 22 of these 49 placentas (including all 17 placentas tested by our research group) were sent to the reference laboratory, according to the approved protocol of the Brazilian Ministry of Health. In the end, 10 negative control pregnant women, selected out of the epidemic outbreak and without symptoms of arboviruses infection during gestation, were included and their placentas were also collected according to the protocol of placental sampling used in this study.

### Serum and Urine Sampling

Serum and urine were obtained from all women during gestation at the time they had symptoms compatible with arbovirus infection. Serum was also collected from all pregnant women during childbirth. Urine collection was done in a sterile universal collection vial, while three aliquots of 5 ml of peripheral blood were collected in EDTA tubes for serum separation. One tube of blood and one of urine were sent on ice to the Laboratory of Emerging Virus Studies (LEVE) of Unicamp for qRT-PCR by TaqMan method with probes specific for annealing in ZIKV viral genome. Two other blood bottles were sent to the Adolfo Lutz Institute, a reference laboratory (RL) for arbovirus detection in the State of São Paulo, up to 24 h for serological and/or qRT-PCR detection of ZIKV. In the current study, we retrieved their data on qRT-PCR. Serum was obtained by centrifugation at 3000 rpm for 10 min.

### Placenta Sampling

**FIGURE 2:**
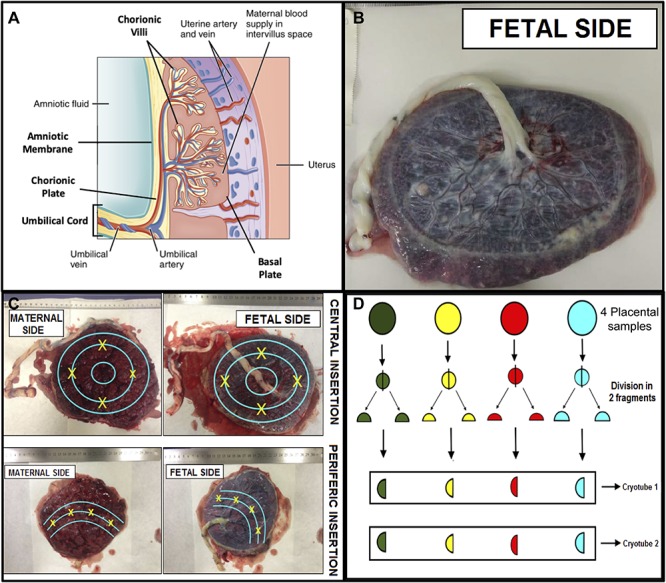
Scheme for systematic placental sampling. **(A)** Cross section of the placenta showing its components – adapted from OpenStax College. 2017. Anatomy and Physiology, p1333, figure 28.11. http://cnx.org. **(B)** Placenta fetal side. **(C)** Points of placental samples collection based in the umbilical cord insertion. **(D)** Sample processing and storage.

The collection of placental material was performed in a systematic manner, with the shortest interval after delivery, in order to obtain a comprehensive sampling of four fragments equidistant to cord insertion containing all regions of this tissue (Figure 2), avoiding areas of visible calcification, hematoma, or infarct. Sampling was initiated from the basal plate through the chorionic villus and chorionic plate, as well as the umbilical cord and amniotic membrane (Burton et al., 2014).

Four fragments about 1.5 × 1.5 cm and about 5 mm thick were cut, which were then divided into two fragments, one 1.0 × 1.0 cm and the other 0.5 × 0.5 cm. The 1 cm^2^ fragment was fixed in 10% formaldehyde solution, and the 0.5 × 0.5 cm fragments were further divided into two fragments and frozen in liquid nitrogen with subsequent storage at −80°C. Each Eppendorf contained four pieces from all different areas, in order to ascertain representativeness. RNA extraction was performed and further detection of ZIKV was performed by qRT-PCR. The same procedure was performed for the other regions of the placenta, including the amniotic membrane, chorionic villi, and chorionic plate (Figure 2). For the umbilical cord samples, we collected three fragments containing the full thickness of the tissue. All material was stored in the Institutional Biobank of the Women’s Hospital.

In addition, three fragments of 1 cm^3^ of the placenta of 22 of the 49 childbirths performed at the University of Campinas were shipped in dry ice to the RL for ZIKV detection (collected by the clinical team, not the research team, as part of the institutional protocol, following the national recommendations for healthcare).

### RNA Extraction

The fragments from each of the four placental regions were added in a single tube for TRIzol Plus RNA extraction associated with the PureLink RNA Mini Kit (Invitrogen) for further detection of ZIKV by qRT-PCR. Three umbilical cord samples were collected, one of which was submitted to TRIzol RNA extraction protocol. The results obtained were then compared with the official data of detection of ZIKV in the placenta of these same pregnant women published by the public reference laboratory for arbovirus detection in the State of São Paulo. Finally, to determine if the difference in the detection rate of ZIKV was due to the placental sample collection or the RNA extraction protocols, a fragment of each of the regions was submitted to a new RNA extraction using the RNeasy Mini Kit (Qiagen), following the protocol used at RL. For RNA extraction by TRIzol, four micro fragments from each placental region stored in the biobank were sent to the Emerging Virus Study Laboratory. The material was weighed and macerated in beads of zirconia crystals in the Magna Lyser instrument (Roche Life Science) in 1 ml of TRIzol Plus RNA extraction. The purification was performed by the PureLink RNA Mini Kit (Invitrogen) following a protocol suggested by the manufacturer. The collected tissues were also submitted to RNA extraction using the RNeasy Mini Kit (Qiagen), the same kits used by the Reference Laboratory. In addition, RNA was obtained from 140 μl of serum and urine obtained during the acute phase of the disease and/or time of deliver, after extraction with the QIAamp Viral RNA Mini Kit (Qiagen, Hilden, Germany). All RNA samples were quantified by analyzing the absorbance at 260/280 nm in a Nanodrop One (Thermo) spectrophotometer and stored at −80°C until use.

### qRT-PCR for Detection of ZIKV

The detection of ZIKV by qRT-PCR was performed following a previously published protocol (Lanciotti et al., 2008). Briefly, the cycling conditions were: 45°C for 1 min (RT step); 95°C for 5 min and 45 cycles at 95°C for 15 s; and 60°C for 1 min. Primers and probes for ZIKV were used in the final concentrations of 400 and 200 nM, respectively. All reactions were performed with the TaqMan Fast virus 1-Step Mastermix kit (Applied Biosystems by Thermo Fisher Scientific) in a Quantistudio 3 (Thermo) in reactions with 12 μl final volume, using 7 μl of template RNA (approximately 1 μg). In addition, to ensure the same parameters used by Reference Laboratory, all samples extracted using the QIAamp Viral RNA Mini Kit were also tested for ZIKV by Taqman GoTaq Probe 1-Step qRT-PCR System Kit (Promega). Reactions of qRT-PCR with cycle threshold values (Cq) greater than 40 cycles were considered negative.

### ZIKV Quantitation in Placentas and Urine by qRT-PCR

Zika virus RNA levels in the placenta during childbirth and urine collected during the acute phase of infection were determined by TaqMan one-step quantitative reverse transcriptase PCR (qRT-PCR) using the TaqMan universal PCR master mix (Thermo) and expressed on a log_10_ scale as viral RNA equivalents per gram or per milliliter after comparison with a standard curve produced using serial 10-fold dilutions of ZIKV RNA obtained from a purified and tittered ZIKV Br strain (BeH815744). All reactions were performed using the same conditions and cycling algorithm described above regarding qRT-PCR for detection of ZIKV. The amplification of the Tyrosine 3-Monooxygenase/Tryptophan 5-Monooxygenase Activation Protein Zeta (*ywhaz*) gene was used as a control for normalization and to check the quality of all RNA extracts from placentas, while the amplification of *b-actin* gene was used as a control in urine.

### Anatomopathological Analysis

All placentas have been submitted to a standard Pathology protocol concerning the 2016 Amsterdam Consensus statements (Khong et al., 2016). Hematoxylin–eosin analysis was performed in blocks of the 39 regions positive for ZIKV by qRT-PCR.

### Data Analysis

Comparisons between the frequency of viral detection in different placental sites were performed by Chi-square test of contingency. Comparisons of viral load among different placental regions were done by two-way ANOVA. The correlation between viral load in the urine and in the placental regions and gestational age at diagnosis was tested using the Pearson correlation coefficient. All statistical tests were done using GraphPad Prism version 7 for Mac (GraphPad Software, San Diego, CA, United States). A *P*-value ≤ 0.05 was considered significant.

### Ethical Considerations

The research project followed all rules for the use of human samples, with approval by the Research Ethics Committee of the University of Campinas (UNICAMP), CAAE: 64253416.9.0000.5404. All samples were stored in the Institutional Biobank as approved by the ethics committee of the same hospital, CAAE: 60863516.2.0000.5404. All women signed the informed consent form authorizing the collection, storage, and use of clinical samples.

## Results

### Characteristics of Considered Women During ZIKV Outbreak in Campinas-SP

Seventy-seven pregnant women aged between 16 and 40 years (mean: 26.7 ± 6.5 years) presented symptoms of arbovirus infection during gestation and were referred to the Women’s Hospital at the University of Campinas during 2015/2016. Of these, 49 had childbirth at the institution and 17 had placental samples collected and tested by our group. In addition, 22 of these 49 childbirths (including all 17 placentas tested by our group) tested negative for ZIKV qRT-PCR in the reference laboratory, according to official reports from the Adolfo Lutz Institute (Figure 1). Seven of these cases presented positive testing for ZIKV in serological and/or molecular tests by qRT-PCR during gestation in urine samples in tests carried out at the reference laboratory and/or Laboratory of Emerging Virus Studies Institute of Biology of Unicamp (Figure 1). All the pregnant women were negative for HIV, hepatitis B, hepatitis C, syphilis, toxoplasma, cytomegalovirus, rubella, and Epstein–Barr virus, according to routine tests.

The clinical, demographic, and childbirth characteristics of all pregnant women with and without systematic placental sampling are included in Table 1. Conjunctivitis, exanthema, fever, headache, joint pain, myalgia, and pruritus were the symptoms that characterized the ZIKV-positive patients. The cases with the worst perinatal outcomes were from later infections (27 and 28 weeks and evolved to neonatal death).

**TABLE 1:** Sociodemographic, clinical, obstetrical characteristics, and maternal/fetal outcomes of women with follow-up and childbirth at the hospital at University of Campinas, for suspected ZIKV, among the group with systematic placental sampling (17) and without (32).

**Clinical and demographic characteristics**	**(*n* = 17)**	**(*n* = 32)**
Age (years ± SD)	27.3 ± 6.2	25.9 ± 6.7
Gestational age of onset of symptoms (weeks ± SD)	22.7 ± 8.9	30.1 ± 8.5
Symptoms		
1° trimester	3(18%)	1(3%)
2° trimester	6(35%)	9(28%)
3° trimester	8(47%)	22(68%)
Conjunctivitis	2(12%)	1(3%)
Exanthema	11(65%)	17(53%)
Fever	9(53%)	13(40%)
Headache	6(35%)	15(46%)
Joint pain	5(29%)	6(18%)
Myalgia	3(18%)	9(28%)
Pruritus	8(47%)	12(37%)
Mean interval between onset of symptoms and urine and/or blood sample collection (days ± SD)	12.9 ± 24.4	3.4 ± 3.7
Vaginal delivery	9(53%)	19(60%)
Cesarean delivery	8(47%)	13(40%)
Fetal malformation*	2(12%)^1^	3(9%)^1^
Neonatal death*	2(12%)^1^	2(6%)^1^

*Same cases; ^1^Microcephaly.

Of the 17 placentas analyzed, 14 (82%) were positive for ZIKV genome detection by qRT-PCR assay after RNA extraction using PureLink RNA Mini Kit (Invitrogen) in some of the placental regions. Following the same methodology used by the reference laboratory applied to the systematic collected placental samples, submitted to RNA extraction using the RNeasy Mini Kit (Qiagen), we obtained 10 (59%) positive placentas, in comparison with none reported by the reference laboratory. The region with the highest number of ZIKV detection was the chorionic villi, followed by the chorionic plate, amniotic membrane, basal plate, and umbilical cord (Table 2). There was no correlation considering ZIKV viral load and gestational age at diagnosis of ZIKV acute phase (0.3 ≥ ρ ≥ −0.3 by Pearson correlation coefficient). In order to validate the specificity of the qRT-PCR assay in placental RNA, we included placenta samples of 10 pregnant women without arboviruses infection symptoms during gestation as negative controls. All these samples were negative for ZIKV detection using both protocols of RNA extraction and qRT-PCR assay, including the umbilical cord, chorionic plate, chorionic villi, basal plate, and amniotic membrane.

**TABLE 2:** Results of RT-qPCR for ZIKV detection by placental region (*n* = 17).

**Cases**	**GA**	**UC**	**AM**	**CP**	**CV**	**BP**	**Total**
P1	8	343303.6	236974.8	200549.5	327232.1	1219576.7	5
P2	16	0.0	216071.4	63279120.9	73214.3	0.0	3
P3	27	203448.3	53422.6	38988.1	33333.3	87714.2	5
P4	24	28794.6	0.0	28392.9	37267.1	0.0	3
P5	36	0.0	0.0	0.0	0.0	0.0	0
P6	20	0.0	60000.0	2040.8	105555.5	219387.8	4
P7	16	172899.2	39115.6	0.0	87433.9	0.0	3
P8	27	0.0	0.0	0.0	52232.1	102100.8	2
P9	37	0.0	268989,5	0.0	30952.4	0.0	2
P10	7	0.0	0.0	0.0	73602.5	0.0	1
P11	10	0.0	0.0	0.0	0.0	0.0	0
P12	29	0.0	0.0	0.0	0.0	0.0	0
P13	26	21428.5	0.0	15109.9	0.0	0.0	2
P14	28	0.0	0.0	0.0	10786285.7	0.0	1
P15	30	0.0	0.0	0.0	25595.2	100180.0	2
P16	27	0.0	0.0	NT	6462159.9	18811734.7	2
P17	20	0.0	39610.4	27272.7	9027738.1	33163.2	4

*GA, gestational age at infection in completed weeks, UC, umbilical cord, AM, amniotic membrane, CP, chorionic plate, CV, chorionic villus, BP, basal plate. The values shown are the viral load expressed as viral RNA equivalents of ffu per gram of placenta, NT: not tested or missing sample.*

No correlation was observed between the dissemination of ZIKV in the placenta and the presence of fetal findings. The only two newborns who presented fetal growth restriction and fetal malformation were children of patients P.3 and P.14, who had distinct patterns of placental infection. While the P.3 patient presented positivity for ZIKV in all placental regions, patient P.14 had ZIKV only in the chorionic villi (Table 2).

**FIGURE 3:**
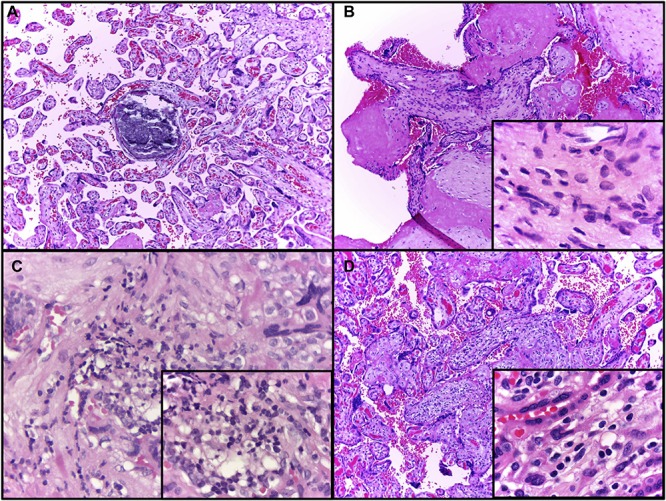
Representative evaluation of placental, hematoxylin–eosin findings. **(A)** Patient 1, dystrophic calcification in a third trimester villus, a very common finding at term (10 × objective) highlighted by the asterisk. **(B)** Patient 2, chorionic villus presenting increased stromal cellularity (10 × objective); in the inset, frequent spindle cells consistent with fibroblasts are seen in the villous stroma (100 × objective). **(C)** Patient 6, Basal plate sample with chronic deciduitis (40 × objective); in the inset (100 × objective), infiltrated lymphocytes, macrophages and plasma cells are visible, denoting a chronic deciduitis at basal plate. **(D)** Patient 6, chronic villitis affecting several villi (10 × objective); in the inset, the villous stroma contains mononuclear cells, as lymphocytes and histiocytes (100 × objective).

According to the statements from the 2016 Amsterdam Consensual for placental sampling and inflammatory and vascular lesions, Table 3 presents the anatomopathological results, and the most common findings were maternal vascular malperfusion lesions (eight), inflammatory lesions (six), including focal low- (four) and high-grade villitides (two), placental hypoplasia or weight under the 10th centile (four), and focal avascular villi (one). Intervillous thrombi, not defined in the Consensus, were present in four patients’ placentas. Hematoxylin–eosin analysis in blocks of the 40 regions sampled from the placenta and positive for ZIKV by qRT-PCR showed that the most common findings were increased cellularity (six regions), villitis (five regions), and calcification (three regions). Some of these findings are represented in Figure 3. Findings from ZIKV-negative cases were not different and mostly non-specific in comparison to the ZIKV-positive cases.

**TABLE 3:** Results of anatomopathological findings and hematoxylin–eosin analysis, according to the 2016 Amsterdam consensus statement, of Zika virus-positive placentas.

**Patient**	**P1**	**P2**	**P3**	**P4**	**P6**	**P7**	**P8**	**P9**	**P10**	**P13**	**P14**	**P15**	**P16**	**P17**	**Total**
**Gross features**															
Adequate weight	+	−	−	−	+	+	+	+	+	+	+	−	+	−	9
Extreme low weight (hipoplasia)	−	+	+	+	−	−	−	−	−	−	−	+	−	+	5
**Microscopic features**															
**Cord**															
Post mortem abnormalities	−	−	−	−	−	−	−	−	−	−	+	−	−	−	1
**Membranes**															
No abnormalities	−	−	−	+	−	−	+	+	+	+	−	−	+	+	7
Maternal vascular malperfusion (decidual vasculopathy)	−	+	+	−	−	+	−	−	−	−	−	−	−	−	3
Meconium deposition	−	+	−	−	+	+	−	−	−	−	−	+	−	−	4
Focal maternal inflammatory response	−	−	+	−	−	−	−	−	−	−	−	−	−	−	1
Other inflammatory lesions (chronic deciduitis)	+	−	+	−	−	−	−	−	−	−	−	−	−	−	2
Post mortem abnormalities	−	−	−	−	−	−	−	−	−	−	+	−	−	−	1
**Villi**															
Adequate maturation	+	+	+	−	+	+	+	+	+	+	−	+	+	+	12
Low-grade chronic villitis	−	−	+	−	−	−	−	+	+	−	−	+	+	−	5
High-grade chronic villitis	+	−	−	−	+	−	−	−	−	−	−	−	−	−	2
Distal villous hipoplasia	−	−	−	+	−	−	−	−	−	−	−	−	−	−	1
Centrally placed infarcts	−	−	−	−	+	−	−	−	−	−	−	−	−	−	1
Excessive perivillous fibrin deposition	−	−	−	−	−	+	−	−	+	+	+	+	−	−	5
Low-grade fetal vascular malperfusion	−	−	−	−	−	−	−	+	−	−	−	−	−	−	1

*The main findings in the three ZIKV-negative placentas of the 17 placentas included in this cases series were: Maternal vascular malperfusion (decidual vasculopathy) and Meconium deposition in the membranes, and excessive syncytial knots for term and excessive perivillous fibrin deposition in villi. These findings were found in only one patient.

**FIGURE 4:**
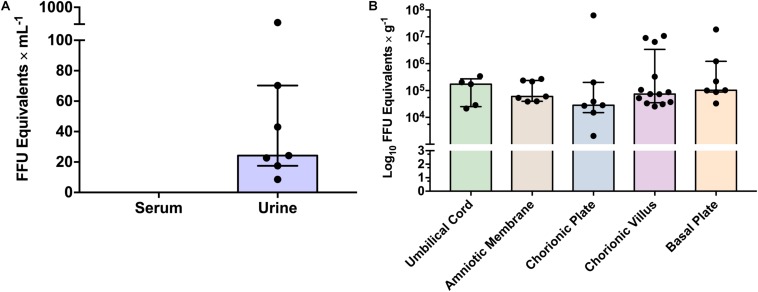
Zika virus viral load in different clinical samples from analyzed patients. **(A)** ZIKV viral load in urine during acute phase by qRT-PCR. Bars represent medians and error bars represent interquartile range. Each point represents an individual patient (*n* = 7). **(B)** ZIKV viral load in different placental regions. Bars represent medians and error bars represent interquartile range. Each point represents an individual patient (*n* = 17). Two-way ANOVA was performed and no statistical significant differences on viral load were observed between placental regions (*p* ≥ 0.05).

The ZIKV load was not statistically different among different placental regions tested, including the basal plate, chorionic plate, chorionic villi, amniotic membrane, and umbilical cord (*p* = 0.533, by two-way ANOVA analysis). In addition, although ZIKV was more frequently detected in the chorionic villi, most likely due to the restricted sample size, there was no statistically significant difference among the frequency of viral detection in the tested placental regions (*p* = 0.073 by chi-square test). Finally, there was no clear association between the viral load in urine during the acute phase and the viral detection in the different placental regions in the same patients, after delivery (0.3 ≥ ρ ≥ −0.3 by Pearson correlation coefficient). Interestingly, one patient presented positivity in all different placental regions but did not present positivity in the urine. Five cases presented positive ZIKV in the placenta, with no positivity during the acute phase testing. Viral quantification in the acute phase in the urine ranged from 10^1^ to 10^3^ FFU eq/ml and in the different placental regions from 10^3^ to 10^8^ FFU eq/g (Figure 4).

## Discussion

Detection analyses in the various biological samples used in this study demonstrate differences in positivity according to timing between the samples corresponding to the acute phase (symptoms) and the chronic phase (childbirth). While ZIKV was detected in most patients during the symptomatic acute phase in urine samples, ZIKV was not detected in serum samples during delivery, contrasting with the high frequency of ZIKV detection in placenta. This suggests that the placenta may be a site of replication and persistence of ZIKV months after the disappearance of the symptoms. This fact emphasizes that pregnant women who were underreported for ZIKV could potentially present CZS, raising the degree of disturbance that this pathogen may have generated during the ZIKV epidemic.

The persistence of ZIKV in placenta can be associated with several characteristics of this organ, which allows the mother not to reject the fetus during development (Burton and Jauniaux, 2015; Nelson, 2015). First, the layers of trophoblast, syncytiotrophoblast, and fetal endothelium in the chorionic villi act as a blood–placental barrier that promotes local immunotolerance, in part due to the action of syncycines (Mi et al., 2000; Frendo et al., 2003). Second, there are in placenta a large flow of blood and maternal circulating immune cells (Klase et al., 2016). Thus, placenta is a site for viral persistence of different viruses, such as rubella virus.

Another relevant finding of our study was the discrepancy between our qRT-PCR results and the results presented by the reference laboratory. In addition to the highest sensitivity test, we found 23% more cases using the Invitrogen RNA extraction kit than the Qiagen kit. We also detected ZIKV in the acute phase through serology in the urine and later in the placenta, where we found RNA in all placental regions including the umbilical cord, with a highlight of the chorionic villus, which showed the highest positivity. More severe fetal involvement was observed in the two mothers who presented clinical symptoms during the third trimester of gestation.

None of the placenta samples tested positive for ZIKV in the national reference laboratory in comparison to 82% of positive samples in the systematic study samples. We cannot rule out the effect of postponing placenta sampling and inadequate storage as explaining the disparity between our results and those of the reference laboratory. Our results emphasize the importance of collecting the placenta shortly (within 12 h) after delivery and adequately storing it.

A greater detection sensitivity of the Invitrogen Kit was identified, with 14 positive patients compared to the 10 positive samples diagnosed by the Qiagen Kit performed at our lab, but none was positive at the reference lab. The placental samples from the same patients sent to the reference lab were not collected by the research team but were obtained through collection at three random regions of the placenta and kept at −20°C, according to the guideline determined by the Ministry of Health of Brazil (Ministério da Saúde, 2015). This result corroborates the importance of systematic collection of the placenta, with adequate storage in the accurate diagnosis of ZIKV by qRT-PCR.

As a limitation, it is important to point out that of the 49 women who delivered at our institution, only 22 were sampled for placental diagnosis (as part of the assistance protocol) and 17 submitted to systematic sampling. This reflects the difficulty of implementing routine procedures involving unplanned deliveries with different teams working in the obstetric room.

It was also found that the detection of ZIKV in the acute phase by qRT-PCR technique of RNA extracted from serum or urine presented less positivity in relation to the detection of placental biopsies. As previously described, the detection window of ZIKV in the acute phase by biological samples, in the case of serum and urine, covers a short period of time, rarely exceeding 2 weeks after the appearance of the first symptoms. Since this period may have been exceeded at the time of collection and serological techniques have higher rates of non-specificity, the diagnosis of this infection may be impaired (Klase et al., 2016). Therefore, even in the absence of viremia or virus in the urine, late detection in placentas collected and stored in a systematized and standardized way is essential for a more accurate viral diagnosis.

Chorionic villi samples showed higher number of positive samples and umbilical cord lower number of positivity for ZIKV, corresponding, respectively, to the regions with the highest and lowest cellularity. However, most likely due to the low number of included cases, such findings were not statistically significant. The chorionic villi in particular is known to be the region with the highest concentration of Hofbauer cells (HCs), which suggests a potential correlation with the transmission of ZIKV and potential congenital disease. There are studies suggesting that HCs can contribute to the spread of the virus in the placenta and promote vertical transmission (Besnard et al., 2014; Jurado et al., 2016). In fact, a detailed placental examination performed recently in three cases of fetal disruption caused by the ZIKV evidenced a hyperplasia of Hofbauer cells with signs of inflammation in this organ (Beaufrère et al., 2019). Thus, the different degrees of positivity for ZIKV in the various placental regions may be directly correlated with the distinct impairment in placental, fetal, and neonatal development and function during pregnancy, in addition to the gestational stage presented.

The most frequent morphological alterations found in our study, such as placental hypoplasia, maternal vascular malperfusion, and maternal–fetal hemorrhage, are non-specific findings, present for example in cases of intrauterine growth restriction (IUGR) and pre-eclampsia (Neto et al., 2011). Inflammatory findings are also frequent even in pregnancies with adequate outcomes (Romero et al., 2018). Moreover, distinction between low- and high-grade villitis is important because high-grade villitis has significant associations with IUGR, neurodevelopmental impairment, and likelihood of recurrence (Tamblyn et al., 2013; Khong et al., 2016). Interestingly, some groups showed that ZIKV-infected placentas have a higher risk for development of pathological anomalies than non-infected placentas (Pomar et al., 2019). Placentomegaly, umbilical artery Doppler anomaly, fibromuscular hypertrophy, acute chorioamnionitis, funisitis, chronic villitis, fibrosis, and the presence of neutrophils in the intervillous space can be frequently observed in ZIKV-infected placentas (Beaufrère et al., 2019; Pomar et al., 2019). In fact, it is not totally clear if placental alterations are risk factors for adverse fetal or neonatal outcomes or if they are associated with maternal–fetal transmission of ZIKV, which is estimated between 7 and 26% (Pomar et al., 2019).

Most reports and series of ZIKV cases in pregnancy show more severe cases in early infection, especially in the first trimester. According to studies by Noronha et al. (2016) and Singh et al. (2016), most infants with CZS were the children of mothers who had symptomatic Zika fever in the first trimester. Miranda-Filho et al. (2016) found that 70% of mothers of children with microcephaly had rashes in the first trimester. In our study, the cases with the worst perinatal outcomes were from later infections (27 and 28 weeks), and both malformed newborns evolved to neonatal death.

One limitation of this study is that we had a very restricted number of malformations among the considered cases to permit further inferences based on viral load, and different regions of placenta showed themselves to be infected. In fact, the expected endemic malformations in consequence of ZIKV were not confirmed in the geographic region. Reasons for the discrepancy between the high incidence of cases in Northeast of Brazil and other regions in the same country or other countries are not fully understood (Instituto de Pesquisa Econômica Aplicada [IPEA], 2018), but can represent different gene polymorphisms, socioeconomic conditions, environmental factors that influence the intestinal microbiota, and the individual history of flavivirus infections.

This study provides further support for viral persistence in the placenta, even in the placenta of pregnant women without ZIKV detected during acute infection or who have had children without CZS. In addition, systematic collection and storage of the same directly promoted the diagnosis of ZIKV. Future studies should address the long-term follow-up of cases with positive placental testing for ZIKV and no diagnosed perinatal adverse outcome.

## Conclusion

Zika virus can infect different regions of the placenta such as the umbilical cord, amniotic membrane, chorionic plate, chorionic villus, and basal plate of infected pregnant women. The discrepancy between the rate of ZIKV detection in the placenta observed in our results and the official data released by the Adolfo Lutz Institute showed that adequate placenta sampling early after birth and snap freezing the collected tissues upon storage at −80°C could be a better method than that used by the reference lab for placenta tissue collection and storage upon PCR testing. In the end, the detection of ZIKV in the placenta after several months of initial symptoms, even in mothers without detection of ZIKV by qRT-PCR in urine and/or serum during the acute phase, suggests that this tissue may be a site for viral persistence during pregnancy.

## Zika-Unicamp Network

Members of the Faculty of Food Engineering, University of Campinas, Campinas, Brazil, Glaucia Maria Pastore. Department of Obstetrics and Gynecology, School of Medical Sciences, University of Campinas, Campinas, Brazil, Helaine Maria Besteti Pires Mayer-Milanez, Carolina C. Ribeiro-do-Valle, Roseli Calil, João Renato Bennini Junior, and Giuliane Jesus Lajos. Clinical Pathology Department, School of Medical Sciences, University of Campinas, Campinas, Brazil, Maria Luiza Moretti, Mariangela Ribeiro Resende, Márcia Teixeira Garcia, Rodrigo Nogueira Angerami and Kleber Yotsumoto Fertrin. Pediatric Immunology, Center for Investigation in Pediatrics, Faculty of Medical Sciences, University of Campinas, Campinas, Brazil, Marcos Tadeu Nolasco da Silva. Department of Human Development and Rehabilitation, Faculty of Medical Sciences, University of Campinas, Campinas, Brazil, Ana Carolina Coan. Neurology Department, Faculty of Medical Sciences, University of Campinas, Campinas, Brazil, Maria Francisca Colella-Santos. Campinas Department of Public Health Surveillance, Campinas, Brazil, Andrea Paula Bruno von Zuben and André Ricardo Ribas Freitas. Department of Genetics, Evolution, Microbiology and Immunology, Institute of Biology, University of Campinas, Campinas, Brazil, Marco Aurélio Ramirez Vinolo. School of Pharmaceutical Sciences, University of Campinas, Campinas, Brazil, Rodrigo Ramos Catharino. Department of Genetics, Evolution, Microbiology and Immunology, Institute of Biology, University of Campinas, Campinas, Brazil, Fábio Trindade Maranhão Costa, Clarice Weis Arns, Matheus Martini and Évellyn Ribeiro de Morais.

## Data Availability Statement

The datasets generated for this study are available on request to the corresponding author.

## Ethics Statement

The studies involving human participants were reviewed and approved by the Research Ethics Committee of the University of Campinas (UNICAMP). The patients/participants provided their written informed consent to participate in this study.

## Author Contributions

The collaborations between all authors were essential for sampling, generate results and helped in developing the idea. EV, GN, APS, PP, DT-T, and JF were responsible to perform the all experiments. JG, RJ, RP, EA, and MC were responsible for follow patients and placental sampling. AA-T, AS, AA, and SC did all morphological and anatomopathological analysis. EV, JP-M, and MC structured the manuscript and did the majority of the writing. All authors revised the manuscript prior to submitting.

## Conflict of Interest

The authors declare that the research was conducted in the absence of any commercial or financial relationships that could be construed as a potential conflict of interest.
